# “Mix-Then-On-Demand-Complex”: *In Situ* Cascade Anionization and Complexation of Graphene Oxide for High-Performance Nanofiltration Membranes

**DOI:** 10.1021/acsnano.0c08308

**Published:** 2021-02-15

**Authors:** Xiaoting Li, Yanlei Wang, Jian Chang, Hao Sun, Hongyan He, Cheng Qian, Atefeh Khorsand Kheirabad, Quan-Fu An, Naixin Wang, Miao Zhang, Jiayin Yuan

**Affiliations:** † Beijing Key Laboratory for Green Catalysis and Separation, College of Environmental and Energy Engineering, Beijing University of Technology, Beijing 100124, P. R. China; ‡ Department of Materials and Environmental Chemistry, Stockholm University, Stockholm 10691, Sweden; § Beijing Key Laboratory of Ionic Liquids Clean Process, State Key Laboratory of Multiphase Complex Systems, Institute of Process Engineering, Chinese Academy of Sciences, Beijing 100190, P. R. China

**Keywords:** graphene oxide, ionic complexation, nanofiltration, poly(ionic liquid), confinement effect

## Abstract

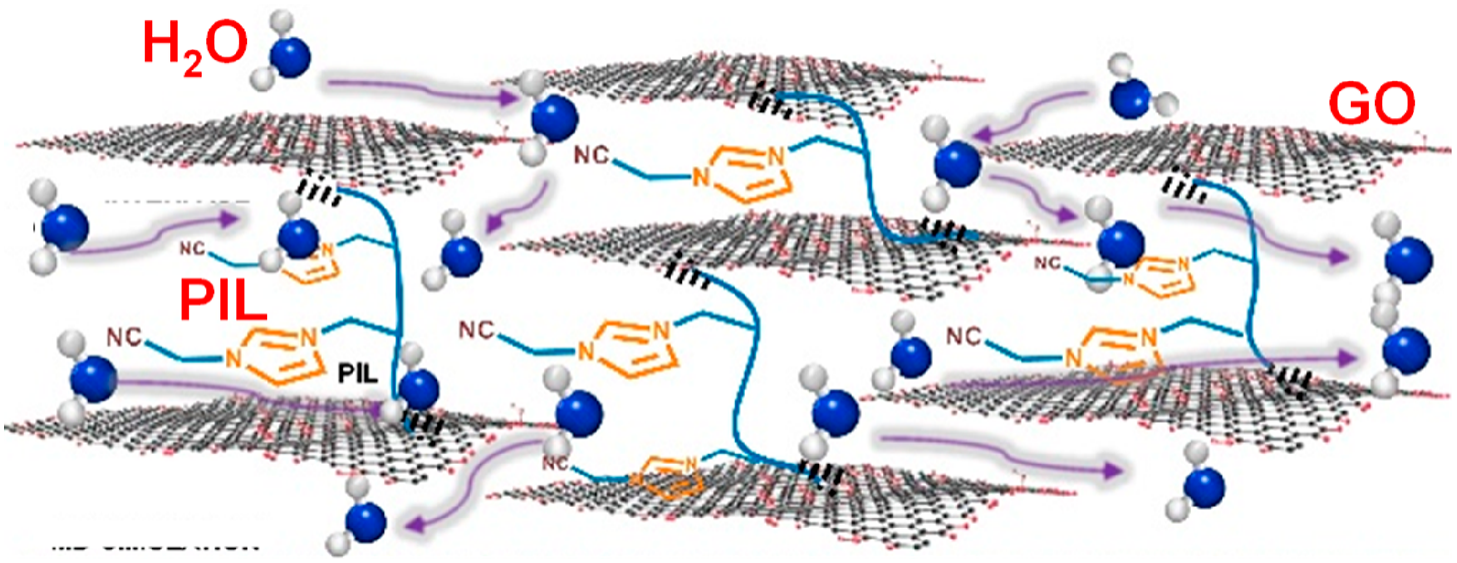

Assembling two-dimensional (2D) materials by polyelectrolyte often suffers from inhomogeneous microstructures due to the conventional mixing-and-simultaneous-complexation procedure (“mix-and-complex”) in aqueous solution. Herein a “mix-then-on-demand-complex” concept *via* on-demand *in situ* cascade anionization and ionic complexation of 2D materials is raised that drastically improves structural order in 2D assemblies, as exemplified by classical graphene oxide (GO)-based ultrathin membranes. Specifically, in dimethyl sulfoxide, the carboxylic acid-functionalized GO sheets (COOH–GOs) were mixed evenly with a cationic poly(ionic liquid) (PIL) and upon filtration formed a well-ordered layered composite membrane with homogeneous distribution of PIL chains in it; next, whenever needed, it was alkali-treated to convert COOH–GO *in situ* into its anionized state COO^–^–GO that immediately complexed ionically with the surrounding cationic PIL chains. This “mix-then-on-demand-complex” concept separates the ionic complexation of GO and polyelectrolytes from their mixing step. By synergistically combining the PIL-induced hydrophobic confinement effect and supramolecular interactions, the as-fabricated nanofiltration membranes carry interface transport nanochannels between GO and PIL, reaching a high water permeability of 96.38 L m^–2^ h^–1^ bar^–1^ at a maintained excellent dye rejection 99.79% for 150 h, exceeding the state-of-the-art GO-based hybrid membranes. The molecular dynamics simulations support the experimental data, confirming the interface spacing between GO and PIL as the water transport channels.

Water of satisfactory quality is essential for our society ranging from food and pharmaceutics to textiles and agriculture, but the shortage and geographical inhomogeneous distribution of clean water is moving into the next global crisis.^1−3^ To tackle this pressing issue, membrane separation technologies stand out because of comparably low energy cost, easy operation, and high efficiency.^4−8^ Recently, ultrathin membranes derived from two-dimensional (2D) materials, particularly graphene oxide (GO) and MXene, have emerged for water treatment, such as desalination, water purification, and wastewater reuse. It is common knowledge that GO-based membranes assembled in a distinctive layered configuration exert a strong size sieving effect for separation; *i.e.*, molecular transport proceeds through both the plane-to-plane nanochannels and in-plane slitlike nanopores.^9−11^ However, water as a Lewis base can nucleophilically attack GO of different oxygenation degrees and weakens the plane-to-plane and plane-to-substrate interaction, causing unfavorable interlaminar swelling of GO sheets and then their peeling off from the underlying porous substrate.^12−16^ To counteract this dilemma, incorporation of polyelectrolytes or similar ionic species as interlayer spacers has been proposed and conducted to engineer multiple interactions to bind adjacent GO layers, which can advantageously modulate the interlayer spacing by polyelectrolytes to tailor the molecular transport for task-specific filtrations.^17−20^

Conventional polyelectrolytes, *e.g*., poly(styrenesulfonate sodium salt) (PSSNa) and poly(diallyldimethylammonium chloride) (PDADMAC), are dominantly water-soluble, so that their processing techniques are water-based.^21,22^ In water, GO sheets that are produced from popular oxidation methods are intrinsically anionic due to the fully or partially dissociable surface carboxylate functionalities and are thus dispersible in a single- or several-layer state due to electrostatic repulsion.^23−26^ Assembling such GO sheets by cationic polyelectrolytes through ionic complexation is a routine conducted by mixing both in water under agitation to reduce structural inhomogeneity, especially at a microscopic scale, which nevertheless often exists in the complexation products. The reason is that inter-polyelectrolyte complexation or polyelectrolyte–GO ionic complexation is entropy-driven and diffusion-controlled, meaning that GO and polyelectrolytes in water must diffuse to meet and then immediately complex each other to form aggregates practically in one step (“mix-and-complex”). As diffusion is kinetically driven by the concentration gradient that changes dynamically along the entire complex process, the ionic aggregates generated at different reaction stages will actually experience varied microenvironments and are (micro)structurally heterogeneous. To counteract such a detriment, the electrostatic layer-by-layer (LbL) assembly of GO and polyelectrolytes and dip-coating polyelectrolytes that are attached on the surface of prestacked GO sheets were used;^27,28^ these methods can be however labor-/time-intensive and require carefully designed conditions that are difficult to scale up. In this circumstance, researchers are urged to alternative methods to assemble GO by polyelectrolytes in a better controllable way.

Recently, poly(ionic liquid)s (PILs) carrying a significantly wider scope of physical and chemical property window than conventional polyelectrolytes have emerged as innovative ionic polymers. A striking feature of PILs is their adaptive solubility in many solvents, meaning that a much larger library of solvents are available for materials processing.^29−31^ In this context, inter-polyelectrolyte complexation between a cationic PIL and poly(acrylic acid) (PAA) has been popularized lately to make functional polymer materials.^32−34^ Given their ionic conductivity, structural flexibility, and high thermal and chemical stability, PILs are rising rapidly as an emerging class of membrane materials for separation processes, *e.g*., gas separation^35−37^ and nanofiltration.^38,39^

To address the “mix-and-complexation” problem in the traditional GO assembly by polyelectrolyte, herein, we introduced a PIL-assisted “mix-then-on-demand-complex” approach *via* separating the complexation step completely from the mixing step. In this concept, the spatial ordering of GO nanosheets and PIL is performed *via* a simple physical mixing in polar aprotic solvent and the subsequent pressure-driven deposition into solid-state thin membranes, which could reach high accuracy due to the absence of ionic complexation between GO nanosheets and PIL in this mixing step; next, the on-demand complexation occurs to ionically lock GO nanosheets in their already desirably ordered state, rather than in the randomly aggregated state as in the conventional “mix-and-complex” approach. This way, the packing and the ionic cross-linking of GO nanosheets are chronologically well-separated, so both can be handled independently to minimize their interference. Note that the tunable counteranions and the cation structure of PILs exhibited a crucial influence on facilitated water transport in the layered confinement environment. This conceptual breakthrough circumvents the diffusion-related structural inhomogeneity issue in previous studies so that better structural control of GO stacking can be exerted during the complexation step. As such, top permeance and high rejection as well as superior durability in water treatment by nanofiltration are obtained.

## Results and Discussion

### Fabrication of COO^–^–GO@PIL**Tf_2_N**–AT Membranes

**Figure 1:**
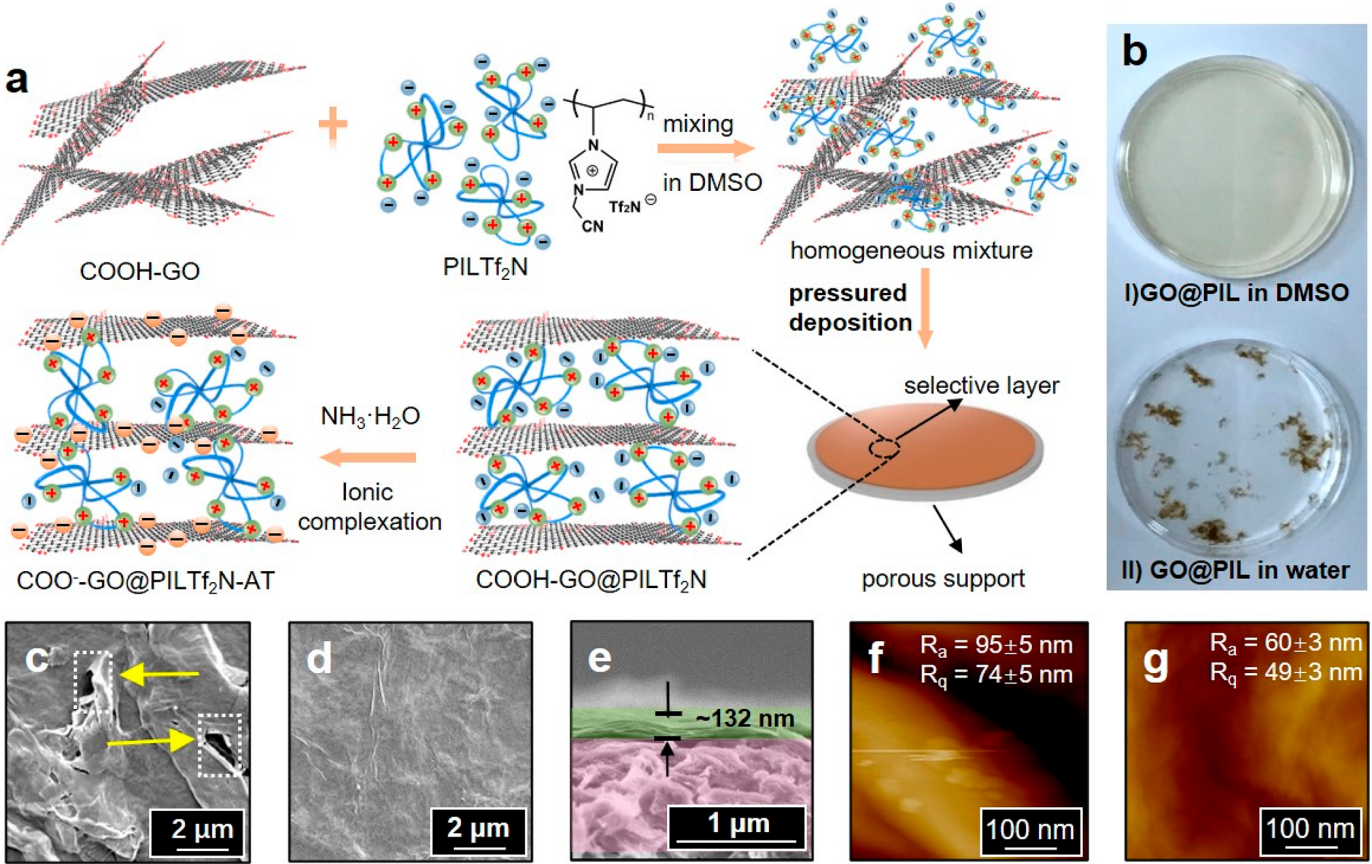
(a) Schematic illustration of the design and fabrication process of a GO/PIL composite membrane COO^–^–GO@PIL**Tf**_**2**_**N**–AT that is deposited on a porous nylon-66 polymer support. First, COOH–GO sheets and the cationic PIL**Tf**_**2**_**N** were mixed homogeneously in DMSO; the mixture was deposited onto the porous support *via* a pressure-assisted filtration-deposition method. Finally, the COO^–^–GO@PIL**Tf**_**2**_**N**–AT composite membrane was alkali-treated by an aqueous NH_3_ solution to deprotonate and anionize the COOH–GO sheets into COO^–^–GO that subsequently complexes *in situ* with surrounding PIL**Tf**_**2**_**N** chains. (b) Photograph of the mixture dispersions of (I) COOH–GO and PIL**Tf**_**2**_**N** in DMSO and (II) COOH–GO and PIL**Br** in water. The GO and PIL concentrations are 0.4 and 0.1 mg/mL, respectively. (c, d) Surface SEM images of the COO^–^–GO@PIL–AT and the COO^–^–GO@PIL**Tf**_**2**_**N**–AT, respectively. (e) Cross-sectional SEM image of the COO^–^–GO@PIL**Tf**_**2**_**N**–AT prepared in DMSO. (f, g) Surface AFM images of the COO^–^–GO@PIL**Br**AT and the COO^–^–GO@PIL**Tf**_**2**_**N**–AT; *R*_a_ ∼ average roughness, *R*_q_ ∼ the root-mean-square average of roughness.

The membrane fabrication procedure is illustrated in Figure 1a. Carboxylic acid-functionalized GO sheets (COOH–GO), produced by a modified Hummers method, and a hydrophobic cationic PIL poly[3-cyanomethyl-1-vinylimidazolium bis(trifluoromethane sulfonyl)imide] (termed PIL**Tf**_**2**_**N**, where **Tf**_**2**_**N** denotes the counteranion) were used as the ionic deposition pair of building units. Chemical structure and characterization details of PIL**Tf**_**2**_**N** can be found in Figure S1. As control experiments, a hydrophilic thus water-soluble cationic one, PIL**Br** (chemical structure in Figure S1) with the same polymer backbone as PIL**Tf**_**2**_**N** but a different counteranion, was used. As shown in Figure S2, the PIL**Tf**_**2**_**N** (ζ potential: +10.2 mV) and PIL**Br** (ζ potential: +4.0 mV) are positively charged in DMSO.

First, a stable dispersion of COOH–GO was prepared in DMSO (Figure S3). The content of COOH in COOH–GO was calculated to be 5.0 wt % according to the X-ray photoelectron spectroscopy analysis (XPS, Figure S4). To note, COOH–GO in DMSO is protonated with little-to-no charge (−1.8 mV, Figure S5). By contrast, in deionized water, due to partial deprotonation, COOH–GO is intrinsically negatively charged, performing like a “polyanion” (ζ potential: −26 mV, Figure S5); consequently, when mixing COOH–GO and PIL**Br** in water, due to strong Coulombic attraction, a “mix-and-complex” event occurs immediately, and insoluble aggregates appear (Figure 1bII).

To circumvent this aggregation issue, we propose the “mix-then-on-demand-complex” concept to clearly set apart the “mix” and “complex” steps. That is, we first mix COOH–GO and a cationic polyelectrolyte evenly; then, whenever needed, *via* alkali treatment we anionize COOH–GO *in situ* into COO^–^–GO that subsequently complexes with the surrounding polycations to form membranes. A key to realize this concept is the GO/polyelectrolyte mixing step in an aprotic solvent that suppresses the deprotonation of COOH–GO, meanwhile keeping its colloidal stability for a more convenient colloidal assembly. Here, DMSO was chosen.

The two individually prepared systems, *i.e.*, COOH–GO and PIL**Tf**_**2**_**N** in DMSO, were mixed into an even dispersion without the occurrence of optical aggregates (Figure 1bI) due to the absence of strong ionic attraction between the neutral COOH–GO and the cationic PIL**Tf**_**2**_**N** in DMSO. Note that still some PIL**Tf**_**2**_**N** chains are associated weakly with COOH–GO through supramolecular interactions, including hydrogen bonding, π–π, and cation−π interaction. Next, pressure-assisted deposition filtration of the mixture dispersion was carried out onto a commercial nylon-66 porous support (Figure S6) with a uniform pore size of 200 ± 30 nm. This pore size is large enough to filter off the free soluble PIL**Tf**_**2**_**N** chains but small enough to block the passage of COOH–GO that has a lateral size of 1.01 ± 0.25 μm (Figure S7). The pressured filtration stacks COOH–GO sheets parallel onto each other on the flat porous support. Note that PIL**Tf**_**2**_**N** chains associated weakly with COOH–GO through supramolecular interactions will be also favorably trapped into stacked GO sheets to form the intermediate membrane state, termed COOH–GO@PIL**Tf**_**2**_**N**. The trapped hydrophobic PIL**Tf**_**2**_**N** chains can effectively manipulate GOs’ plane-to-plane distance and in addition endow the membrane with extra GO-PIL channels, on top of the common GO–GO nanochannels, *i.e.*, membranes of dual transport channels, as discussed in detail later.

In a final step, the resultant COOH–GO@PIL**Tf**_**2**_**N** intermediate membrane was annealed in an aqueous alkali solution at pH = 11 to form the final membrane product COO^–^–GO@PIL**Tf**_**2**_**N**–AT (“AT” stands for “alkali treatment”). In practice, a 0.5 wt % aqueous NH_3_ solution (pH ∼ 11) was used. The alkali treatment neutralizes the −COOH group into COO^–^, and the *in situ* formed polyanion-like COO^–^–GO immediately complexes with PIL**Tf**_**2**_**N** cationic chains that are already positioned around GO in the film. To note, the step of *in situ* cascade anionization and complexation triggered by alkali treatment is chronologically separated from the GO/PIL**Tf**_**2**_**N** mixing step and can be conducted any time on demand.

In typical GO-based separation membranes, molecular transport is strongly dependent on the stacking order of GO sheets. In this study, COOH–GO sheets of an average thickness of 0.9 ± 0.3 nm were used, as measured by atomic force microscopy (AFM) images (Figure S7). As mentioned above, when mixed in aqueous solution, COOH–GO and PIL**Br** instantaneously complex for charge neutralization into insoluble aggregates (Figure 1bII), whereas mixing COOH–GO and PIL**Tf**_**2**_**N** in DMSO ends up with a homogeneous dispersion (Figure 1bI). Such COOH–GO/PIL**Br** aggregates formed in water were sonicated into a suspension and deposited onto the porous nylon-66 support *via* filtration. Its subsequent alkali treatment formed a composite membrane termed COO^–^–GO@PIL**Br**–AT that acts as a “mix-and-complexation” reference membrane.

Top-view scanning electron microscopy (SEM) images of the surface morphology for the reference membrane COO^–^–GO@PIL**Br**–AT (Figure 1c) and our product membrane COO^–^–GO@PIL**Tf**_**2**_**N**–AT (Figure 1d), prepared at a COOH–GO concentration of 10 mg/L in DMSO and a polymer loading content of 25 wt % (with regard to GO), are recorded and analyzed. Larger wrinkles formed by edges of GO sheets and more obvious corrugations were observed in COO^–^–GO@PIL**Br**–AT, indicating its nondense, irregular surface with large holes as defects (Figure 1c). Such defects could adversely flow both water and guest molecules without sufficient separation. In comparison, the COO^–^–GO@PIL**Tf**_**2**_**N**–AT in Figure 1d exhibited a much smoother surface, where wrinkles were much fewer and smaller than those of COO^–^–GO@PIL**Br**–AT, indicative of fewer membrane defects. In addition, the cross-sectional SEM image of COO^–^–GO@PIL**Tf**_**2**_**N**–AT with an average thickness of 132 ± 20 nm shows a well-packed 2D lamellar structure (Figure 1e). Consistent with the SEM observations, the AFM images of COO^–^–GO@PIL**Br**–AT and COO^–^–GO@PIL**Tf**_**2**_**N**–AT in Figure 1f,g further confirm that the membrane surface roughness drops from 95 ± 5 to 60 ± 3 nm, respectively. These results qualify COO^–^–GO@PIL**Tf**_**2**_**N**–AT as an ultrathin, uniform, integrated composite membrane on the support, which is then investigated in detail.

### Microstructures and Molecular Transfer Mechanism of COO^–^–GO@PIL**Tf_2_N**–AT Membranes

**Figure 2:**
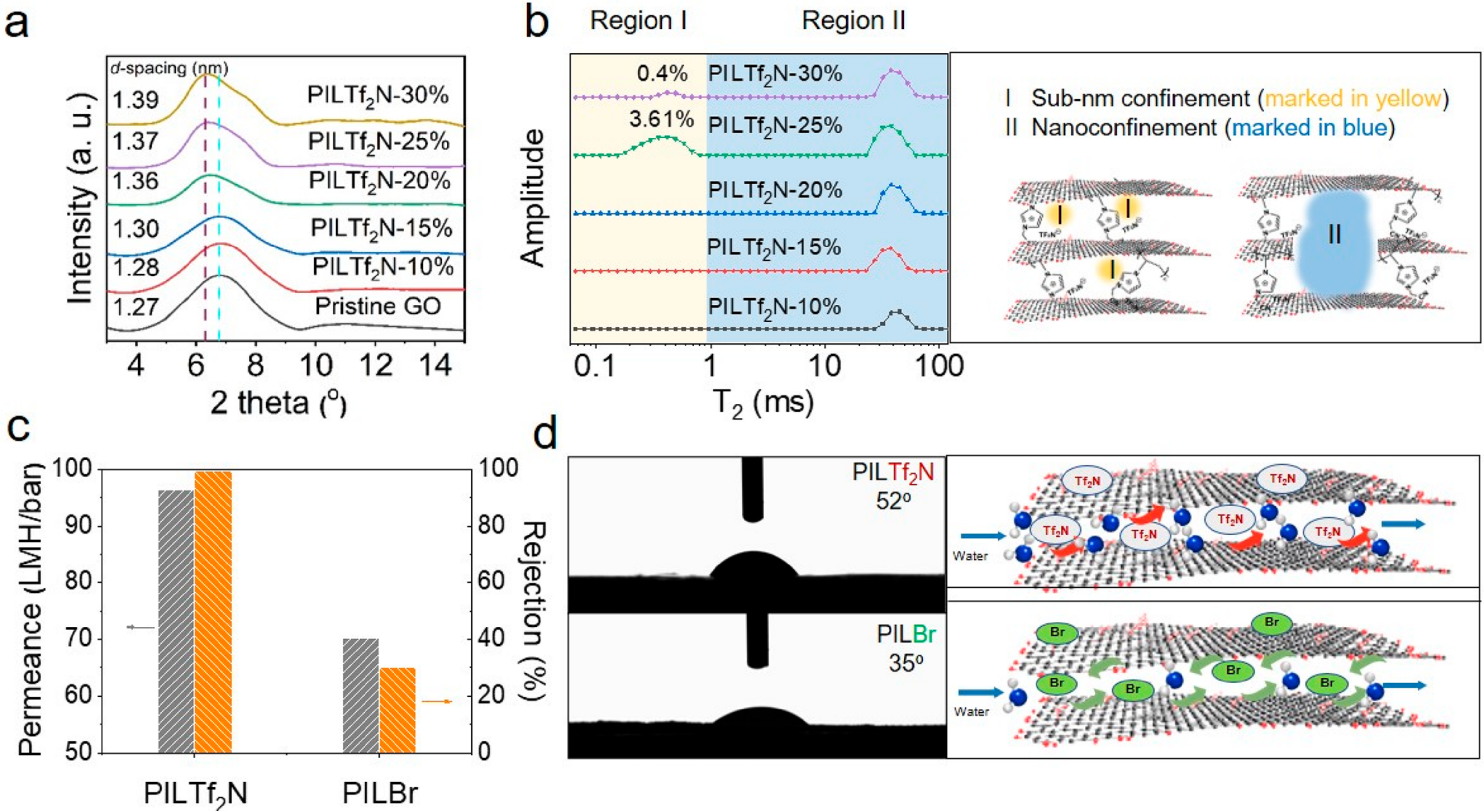
(a) XRD patterns of the pristine GO and the COO^–^–GO@PIL**Tf**_**2**_**N**–AT at different loads of PIL**Tf**_**2**_**N**. (b) Low-field NMR spectra of COO^–^–GO@PIL**Tf**_**2**_**N**–AT at different loads of PIL**Tf**_**2**_**N** and the schematic illustration of confinement space within the COO^–^–GO@PIL**Tf**_**2**_**N**–AT. (c) Comparison of membrane separation performance of COO^–^–GO@PIL**Tf**_**2**_**N**–AT and COO^–^–GO@PIL**Br**AT. (d) Water contact angle of COO^–^–GO@PIL**Tf**_**2**_**N**–AT and COO^–^–GO@PIL**Br**–AT and schematic illustration of the role of counterions in water transport.

To pinpoint the key role of PIL**Tf**_**2**_**N** in the composite membrane, we investigated the interlayer spacing (*d*-spacing) of composite membranes after impregnation of PIL**Tf**_**2**_**N** at different concentrations (Figure 2a). Our initial nanofiltration tests indicated that COO^–^–GO@PIL**Tf**_**2**_**N**–AT could exclude Evans blue molecules while still flowing water through, which speaks of a molecular sieving mechanism. By modulating the PIL**Tf**_**2**_**N** content, expectedly the 2D nanochannels in the composite membranes were observed to vary. As measured by X-ray diffraction (XRD) in Figure 2a, the *d*-spacing of pristine COOH–GO sheets without PIL was 1.27 nm in a wet state; it was enlarged to 1.28, 1.30, 1.36, 1.37, and 1.39 nm when using a PIL**Tf**_**2**_**N** content of 10, 15, 20, 25, and 30 wt % (with regard to COOH–GO) for membrane fabrication, respectively. Furthermore, PIL**Tf**_**2**_**N** in the composite membranes was observed to broaden the diffraction peaks, because it weakens the precise and regular stacking of GO sheets. Meanwhile, as suggested by AFM analysis (Figure S8), the membrane surface roughness increases with increasing PIL**Tf**_**2**_**N** content due to a dewetting effect of the hydrophobic PIL**Tf**_**2**_**N** on the GO surface.

Apart from the *d*-spacing, the chemical environment of water transport channels in COO^–^–GO@PIL**Tf**_**2**_**N**–AT could be effectively tailored by the PIL**Tf**_**2**_**N** loading content because the cationic PIL**Tf**_**2**_**N** upon interplane ionic cross-linking with COO^–^–GO sheets can *in situ* fix their nonequilibrium state. Low-field nuclear magnetic resonance (LF-NMR) spectroscopy was used to detect and characterize molecular transport channels in the membrane using water as a probe molecule. The detected lateral relaxation time (T_2_) represents the thermodynamic equilibrium time of water molecules, which reflects the dimension and amount of nanospace. As shown in Figure 2b, there are two peaks separated into two distinct regions. The peak T_2_ in region II (1–100 ms) represents water molecules in the plane-to-plane slit-like pores, as shown in Figure 2b; expectedly it appears in both pristine GO membrane and COO^–^–GO@PIL**Tf**_**2**_**N**–AT. The peak T_1_ in the region I (0.1–1 ms) represents water additionally confined in nanospace inside the PIL-to-GO nanochannels; these selective nanochannels enable water molecules to pass through exclusively and reject other large molecules to achieve a molecular sieving-based separation. When increasing the PIL**Tf**_**2**_**N** content from 10% to 25%, the peak T_1_ increased from close to 0% to 3.61%. At an even higher loading of PIL**Tf**_**2**_**N** at 30%, the area of peak T_1_ decreased, as excessive PIL**Tf**_**2**_**N** chains may unfavorably block transport nanochannels and nanopores. Another adverse effect of a too high PIL**Tf**_**2**_**N** content is that the blocking effect becomes too dominant, so it shrinks the effective size of nanochannels. As seen from Figure S9, water permeance of COO^–^–GO@PIL**Tf**_**2**_**N**–AT composite membranes continuously increased with increasing PIL**Tf**_**2**_**N** content in the range from 10% to 25% and then decreased to 30%.

In addition to the investigation of microstructures, a similar trend in the membrane surface charge density with respect to the PIL**Tf**_**2**_**N** content was observed, as determined by ζ potential measurements (Figure S10). Increasing the PIL**Tf**_**2**_**N** content from 10% to 30% decreases surface ζ potential from −4.7 to −2.3 mV. Considering that the PIL**Tf**_**2**_**N** chains were coated onto the colloidal GO nanosheet surface homogeneously, the surface charge of GO membranes can reflect the charge density inside the composite membranes. Therefore, the PIL**Tf**_**2**_**N** loading content was fixed at 25% in the following research.

In layered COO^–^–GO@PIL**Tf**_**2**_**N**–AT membranes, water molecules permeate through the interconnected nanochannels formed by adjacent GO sheets as well as the confined nanospaces formed by the interaction between PIL**Tf**_**2**_**N** and GO; *i.e.*, dual paths coexist in the interlayer spacing in the membrane. Previous reports confirmed that water molecules passed preferentially through the hydrophobic rather than hydrophilic regions of GO membranes; *i.e.*, the confined hydrophobic domains facilitate water transport. From their separation performance in nanofiltration, the water permeance of GO@PIL**Tf**_**2**_**N**–AT was 96.38 L m^–2^ h^–1^ bar^–1^ with an Evens blue rejection of 99.79%, much higher than the COO^–^–GO@PIL**Br**–AT of 70.22 L m^–2^ h^–1^ bar^–1^ with 30%, respectively (Figure 2c). Correspondingly, the COO^–^–GO@PIL**Tf**_**2**_**N**–AT derived from hydrophobic PIL**Tf**_**2**_**N** and the COO^–^–GO@PIL**Br**–AT derived from hydrophilic PIL**Br** were compared in terms of their physicochemical properties. The water contact angles were measured to be 52° for the former and 35° for the latter (Figure 2d), suggesting that COO^–^–GO@PIL**Tf**_**2**_**N**–AT was more hydrophobic than COO^–^–GO@PIL**Br**–AT, as expected. Except the well-known hydrophilicity/hydrophobicity effect, the bis(trifluoromethane sulfonyl)imide (**Tf**_**2**_**N**) anion possesses a larger molecular dimension than Br^–^ and resulted in a larger interlayer spacing in the final GO composite membrane. From the wet-state XRD data of COO^–^–GO@PIL**Tf**_**2**_**N**–AT and COO^–^–GO@PIL**Br**–AT composite membranes, we learn that the *d*-spacing of COO^–^–GO@PIL**Tf**_**2**_**N**–AT was 1.37 nm, while that of COO^–^–GO@PIL**Br**–AT was 1.31 nm (Figure S11). Therefore, the expanded transport channels by counteranions are also responsible for the increased mass transfer efficiency of molecules. Overall, a more hydrophobic and larger counteranion is helpful to improve permeance. The rather high rejection of Evans blue in COO^–^–GO@PIL**Tf**_**2**_**N**–AT reaches >99%, far beyond the value of only 30% in COO^–^–GO@PIL**Br**–AT, which is in support of the importance of a denser, defect-less membrane for filtration operation. Next, we investigated the membrane thickness varied by fabricating COO^–^–GO@PIL**Tf**_**2**_**N**–AT from the COOH–GO sheets at different concentrations in the mixture dispersion. As shown in Figure S12, the permeance expectedly dropped sharply with increasing concentration of COOH–GO, which is attributed to the increased selective layer thickness and thus enhanced transport resistance (Figure S13). As a trade-off, the rejection sharply increased.

**Figure 3:**
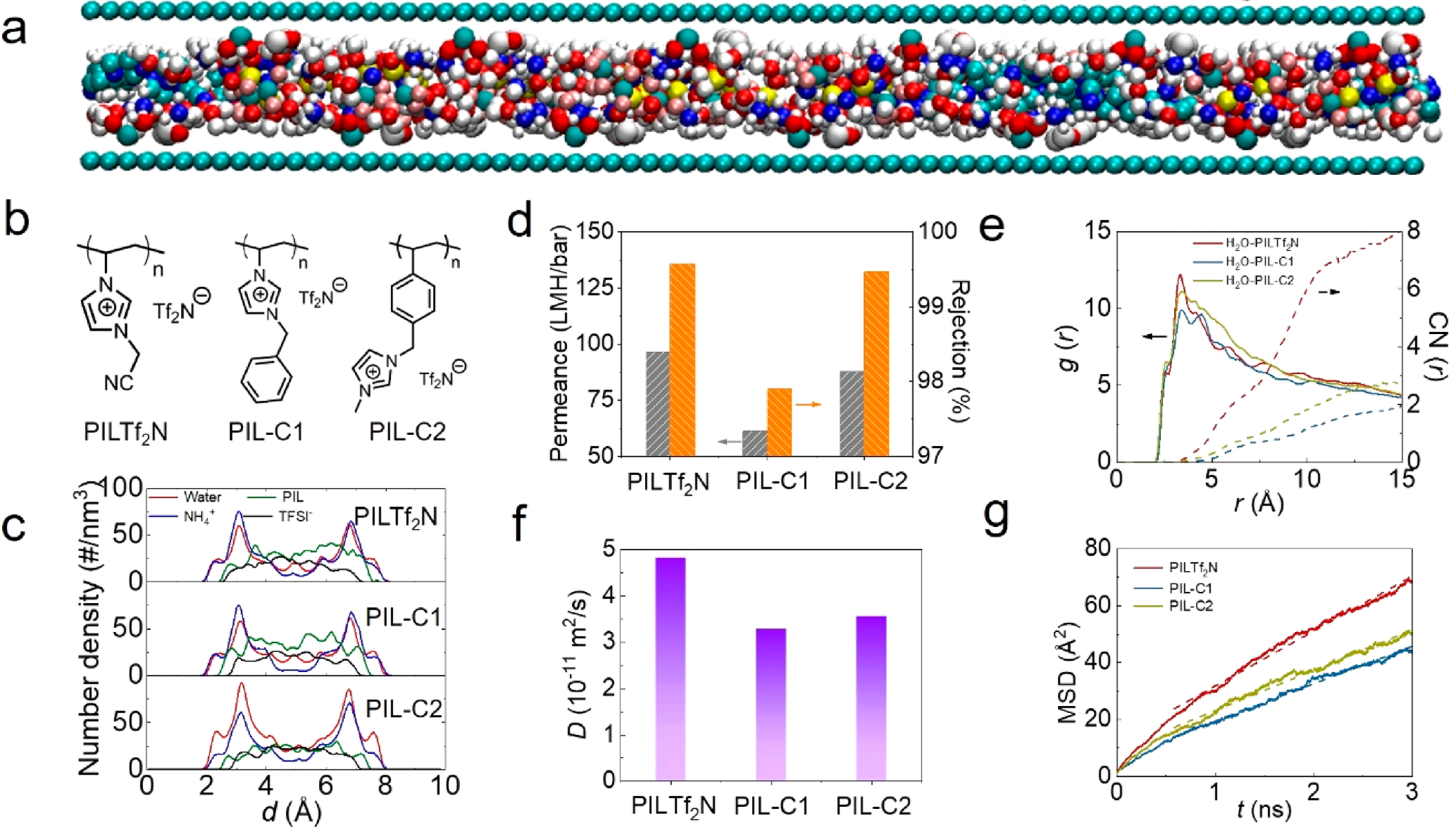
(a) Atomic structure of the nanochannels of the GO@PIL composites in the MD simulation, where cyan, blue, red, white, and yellow represent the carbon, nitride, oxygen, hydrogen, and sulfur atoms, respectively. (b) Three PILs were designed with different backbones and the same counteranion **Tf**_**2**_**N**^–^. (c) Atomic number density distribution of the water, NH_4_^+^, cation, and anion of GO@PIL in the nanochannel. (d) Separation performance of membranes with different PILs in the experiment. (e) Radial distribution function and coordinate number distribution between water and PILs. (f, g) Self-diffusion coefficient of water and mean-square-distance (MSD) in the nanoconfined channel.

Apart from the counteranions, we also designed control PIL polymers to verify the role of polycation backbones in the diffusive transport of water in confined GO nanochannels. PIL**Tf**_**2**_**N** and two control PILs, PIL-C1 and PIL-C2, with the same counteranion **Tf**_**2**_**N** but different backbones built up from different cations (Figure 3b and Figures S14 and S15), were considered here. The molecular dynamics (MD) simulations were conducted to investigate the transport of water molecules in the layered GO nanostructures with impregnated PILs (Figure 3a, Figure S16 and Table S1). As clearly seen from Figure 3c, the PIL cation is located at nearly the entire range of the GO nanoconfined channel, which is attributable to the noncovalent PIL cation–GO interactions and cation–cation solvophobicity force. Two peaks of water molecules are located near the GO walls (where the COO^–^ groups stay), indicating the preferential interactions of water with COO^–^ groups on the GO surface, and presumably a hopping transport mechanism of water molecules along GO–PIL interfacial pathways. As shown in Figure 3b, the only difference between PIL**Tf**_**2**_**N** and PIL-C1 is their terminal substituents, PIL**Tf**_**2**_**N** with the −CN group while PIL-C1 with the benzene ring. The −CN group has a stronger polarity than the benzene ring, thus preferring interaction with water molecules. As for choosing PIL-C2, the distal methylimidazolium cation ring is similar to the cyanomethylimidazolium ring, but PIL-C2 has a larger (polystyrene-like) and more hydrophobic backbone than the PIL**Tf**_**2**_**N** backbone (polyethylene-like). When comparing the nanofiltration performance of these three PILs, apparently the polarity of the distal group of the side chain is more dominant than the backbone, because PIL-C1 shows the lowest flux and rejection, while PIL-C2 has a slightly lower nanofiltration performance than PIL**Tf**_**2**_**N**. Therefore, polymer chains with stronger polar terminal group in the side chains could help reduce the energy barrier of water entrance into the nanochannels. The results of MD simulations confirm that the intensity of the peak in the radial distribution function and the coordinate number distribution between the water and the cation is the highest (Figure 3e). Meanwhile, the PIL as spacer in GO composites contains two ionic components (polymer backbone and counteranions) that can provide a dual hydrophilic/hydrophobic chemical environment in GO interlayer spacing at the same time. As shown in Figure 3f,g, the self-diffusion coefficient of water in the nanoconfined channel and mean-square-distance (MSD) are the highest for COO^–^–GO@PIL**Tf**_**2**_**N** system (4.82 × 10^–11^ m^2^/s). In short, the distal group of the side chain is more dominant in determining the filtration performance than the backbone in the PIL chemical structure.

### *In Situ* Cascade Anionization and Complexation

**Figure 4:**
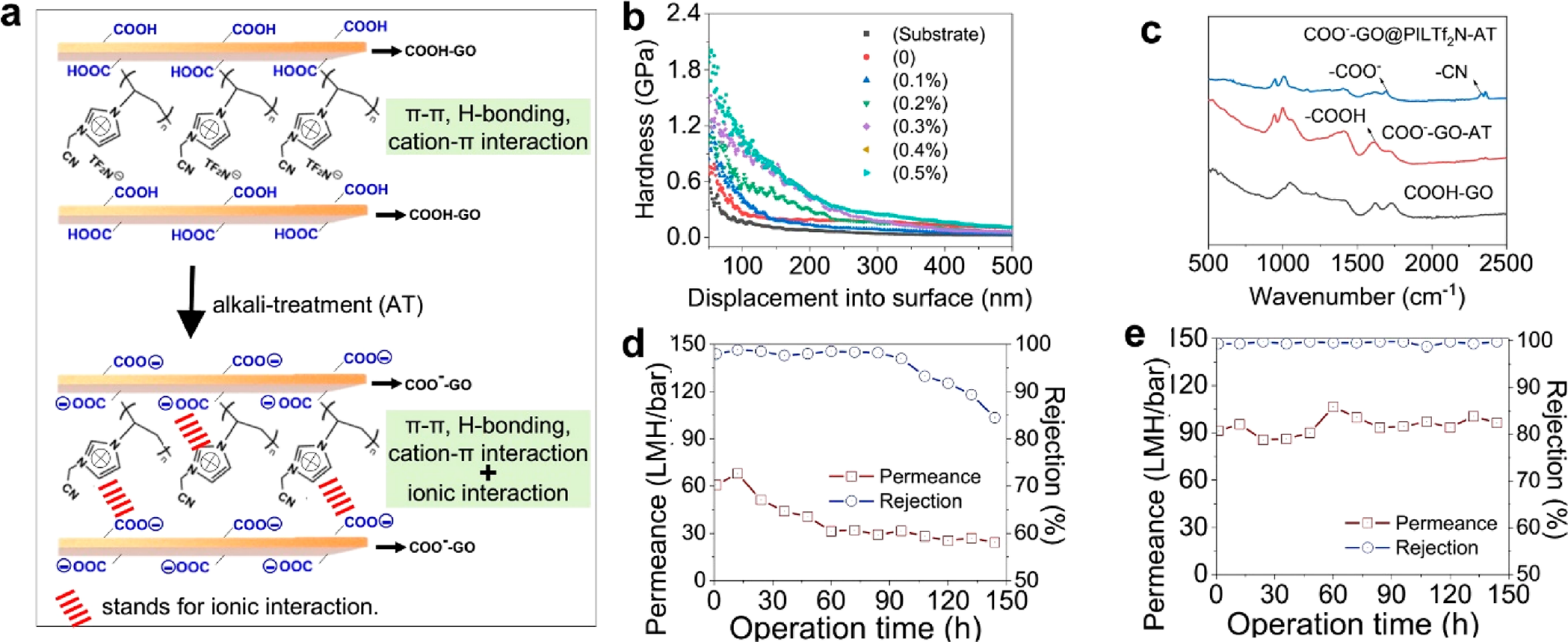
(a) Schematic illustration of *in situ* cascade anionization and ionic complexation of COOH–GO and PIL**Tf**_**2**_**N** assisted by alkali treatment. (b) Results of nanoindentation measurements of pristine COOH–GO and COO^–^–GO@PIL**Tf**_**2**_**N**–AT treated at different concentrations of ammonia. (c) FTIR spectra of COOH–GO (without alkali treatment), COO^–^–GO–AT (alkali-treated), and CO^–^–GO@PIL**Tf**_**2**_**N**–AT(alkali-treated). (d) Nanofiltration stability of COOH–GO@PIL**Tf**_**2**_**N** (without alkali treatment). (e) Nanofiltration stability of COO^–^–GO@PIL**Tf**_**2**_**N**–AT (alkali-treated) by monitoring separation performance under cross-flow and continuous operation up to 150 h. Blue, rejection; red, permeance of water.

Considering applications in water treatment, strong hydration of oxygenated functionalities of GO adsorbs water molecules into the GO interlayer spacing to unfavorably swell GO membranes that varies their separation performance along time. To stabilize the GO-based membrane, the alkali treatment forms strong ionic bonds between COO^–^–GO and PIL**Tf**_**2**_**N** (Figure 4a). Together with supramolecular interactions, such as π–π, cation−π, and hydrogen bonding interactions, the ionic bonds immobilize GO sheets and improve their mechanical properties. As presented in Figure 4b, the COO^–^–GO@PIL**Tf**_**2**_**N**–AT has a hardness of 0.4 GPa, more than twice that of the pristine COOH–GO membrane (0.2 GPa). Meanwhile, the hardness of the membrane is tunable in terms of the NH_3_ concentration, and its effect on the separation performance of COO^–^–GO@PIL**Tf**_**2**_**N**–AT was investigated (Figure S17). When increasing the aqueous NH_3_ concentration, the water permeance was improved from 60.65 to 98.01 L m^–2^ h^–1^ bar^–1^, while the rejection stays above 99% at the NH_3_ concentration of 0.5 wt % or above. The ionic complexation between COOH–GO and PIL**Tf**_**2**_**N** was studied by FT-IR spectroscopy (Figure 4c). The marked adsorption bands at 1700 and 1550 cm^–1^ were ascribed to C=O stretching in COOH and COO^–^ groups, respectively. The weakened strength of COOH groups at 1700 cm^–1^ after NH_3_ treatment confirmed the *in situ* neutralization of —COOH into COO^–^NH_4_^+^. In the nanofiltration tests, the permeance was recorded, typically after 30 min. As shown in Figure S18, the PIL-free pristine COOH–GO membrane reaches a permeance of 9.1 L m^–2^ h^–1^ bar^–1^, COOH–GO@PIL**Tf**_**2**_**N** a permeance of 60.65 L m^–2^ h^–1^ bar^–1^, and COO^–^–GO@PIL**Tf**_**2**_**N**–AT a permeance of 96.38 L m^–2^ h^–1^ bar^–1^ that is the highest and more than 10-fold that of the pristine COOH–GO membrane.

As mentioned above, ionic complexation can resist the swelling of the GO sheets in water. We performed cross-flow and continuous filtration separation processes on membranes to validate their stability and durability in aqueous environments. Figure 4d demonstrates that the water permeance of the COOH–GO@PIL**Tf**_**2**_**N** without alkali treatment (thus with fewer ionic bonds between COOH–GO and PIL**Tf**_**2**_**N**) decreased to 24.26 L m^–2^ h^–1^ bar^–1^, and the rejection drops to 84.48% after 150 h of nanofiltration. In comparison, as shown in Figure 4e, COO^–^–GO@PIL**Tf**_**2**_**N**–AT (with alkali treatment) in the same period maintains excellent rejection (99.79%) and stable permeance (96.38 L m^–2^ h^–1^ bar^–1^). This suggests that alkali treatment is important for the membrane’s antiswelling effect in water. Since molecular selectivity is a pretty important parameter for evaluating nanofiltration membranes, dye molecules were used as molecular probes to test the membrane’s selectivity. We selected a series of dye molecules of similar sizes for this purpose. As shown in Figures S19 and S20, both high rejection and permeance for most dye molecules can be achieved by COO^–^–GO@PIL**Tf**_**2**_**N**–AT. To the best of our knowledge, in comparison to other GO-based hybrid membranes, COO^–^–GO@PIL**Tf**_**2**_**N**–AT exhibited the top separation performance in terms of water permeance and rejection (Figure S21).

## Conclusions

In summary, a “mix-then-on-demand-complex” concept was proposed to better GO-based ultrathin hybrid membranes for nanofiltration assisted by PIL. Meanwhile, by simply manipulating the counteranions and backbone structures of PILs, transport of water can be adjusted in the separation process. This concept is widely applicable to the entire family of 2D materials, as long as they can be functionalized with COOH or other weak acid groups. In addition, the anionization reaction involved in this work is not the only model to trigger *in situ* ionic complexation. For example, cationization chemistry on dialkylamino group-functionalized GO can be coupled with a polyanion to fulfill this concept. This concept is therefore of huge potential in designing 2D material-based membranes, fibers, and beyond.

## Experimental Section

### Materials

Bis(trifluoromethane sulfonyl)imide lithium salt (LiTFSI, 99.95%) was purchased from Io-li-tec. 2-2′-Azobis(2-methylpropionitrile) (AIBN, 98%), 1-vinylimidazole (98%), and bromoacetonitrile (90%) were purchased from Sigma-Aldrich. Natural graphite powder (12 000 meshes) was purchased from Qingdao Huatai Lubricant Sealing S & T Co., Ltd. Aqueous HCl solution, ethanol, DMSO, and THF were purchased from VWR Chemicals BDH. All chemicals were used without any further purification. Solvents were of analytical grade.

### Dispersion of COOH–GO in DMSO

Carboxylic acid-functionalized graphene oxide (COOH–GO) was prepared from natural graphite powder (12 000 mesh) using a modified Hummers method. The as-prepared wet COOH–GO dispersion was treated in a HCl aqueous solution (by diluting 36–38% HCl by 10 times with water) for 12 h and then centrifuged to separate COOH–GO from the HCl solution. The procedure was repeated thrice to remove residual metal ions. Afterward, the COOH–GO sample was washed with anhydrous ethanol in the same way thrice to remove residual HCl aqueous solution. The ethanol-washed GO was separated and air-annealed at 20 °C for 3 h before it was redispersed in DMSO to form a stable dispersion upon a mild sonication treatment. Solvent exchange of ethanol with polar aprotic DMSO provides strong and sufficient solvation forces to stabilize COOH–GO nanosheets in DMSO.^40,41^

### Synthesis of Poly(ionic liquid)s

PIL**Br** and PIL**Tf**_**2**_**N** were synthesized *via* polymerization of the ionic liquid monomers 1-vinyl-3-cyanomethylimidazolium X (CMVImX, X denotes Br and **Tf**_**2**_**N** anions). The monomer CMVIm**Br** was synthesized by reacting 1-vinylimidazole with a 1:1 equimolar bromoacetonitrile in diethyl ether, followed by filtration and vacuum drying to constant weight; the monomer CMVIm**Tf**_**2**_**N** was synthesized *via* anion exchange of the monomer CMVIm**Br** with LiTFSI in aqueous solution.

In the next polymerization procedure, 5 g of CMVIm**Tf**_**2**_**N** and 100 mg of AIBN were dissolved in 50 mL of DMSO. The mixture was deoxygenated by three cycles of the freeze–pump–thaw process. Polymerization started by placing the reaction bottle in an oil bath at 70 °C for 16 h. Afterward, the system was cooled down to room temperature, and the reaction mixture was dropwise added to an excess of THF. The precipitate was redissolved in methanol and precipitated again in THF. Finally, the products were then dried at 90 °C under vacuum overnight. The synthesis of PIL**Br** is similar to that of PIL**Tf**_**2**_**N** except that the monomer CMVIm**Br** was used instead of CMVIm**Tf**_**2**_**N**. The chemical structures of PIL**Tf**_**2**_**N** were confirmed by ^1^H NMR spectra in Figure S1.

The PIL-C1 was synthesized *via* polymerization as follows.

In the first step of monomer synthesis, a mixture of 1-vinylilimidazole (18.82 g, 0.20 mol) and 2,6-di-*tert*-butyl-4-methylphenol (100 mg, 0.45 mol) was dissolved into 40 mL of methanol. Then, benzyl chloride (28 g, 0.22 mol) was added. The reaction was conducted at room temperature for 1 h and then at 60 °C for 1 h. After that, the poly precipitate was obtained by washing with diethyl ether three times and dried *via* high vacuum.

In the next polymerization procedure, a mixture of the above-synthesized monomer (20 g) and AIBN (100 mg, 0.5 wt %) as initiator was dissolved into 80 mL of DMF solution. Then, the mixture was stirred at 70 °C for 24 h under a nitrogen atmosphere. Yellowish precipitates were obtained, and 12 g was received after vacuum drying. A 10 g portion of polymer was first dissolved in 500 mL of deionized water. Then, 100 mL of the aqueous solution of 13 g of bis(trifluoromethane sulfonyl)imide lithium salt was added into the polymer solution. After that, the reaction mixture was further stirred for 2 h, and the precipitate was collected by filtration, washed several times with deionized water, and dried at 60 °C under vacuum.

**PIL-C2** was synthesized *via* polymerization, similar to PIL-C1, only the monomer was different. The monomer synthetic procedure was as follows. A mixture of 1-methylimidazole (30 g, 0.0.35 mol) and 2,6-di-*tert*-butyl-4-methylphenol (100 mg, 0.45 mol) was dissolved in 500 mL of methanol. Then, 4-vinylbenzyl chloride (50.80 g; density, 1.083 g/mL; 46.90 mL; 0.33 mol) was added into the mixture. Then, the reaction was conducted at room temperature for 1 h and then at 40 °C overnight. After that, the precipitate was obtained, washed with diethyl ether three times (1000 mL in total), and dried *via* a high vacuum.

### Polydopamine (PDA) Modification of the Support Surface

Dopamine hydrochloride (2 mg/mL) was dissolved in a tris(hydroxymethyl) aminomethane (THAM) aqueous solution (pH = 8.5, mM), which contains CuSO_4_ (5 mM). A porous nylon-66 flat film was immersed in the THAM buffer solution for 3 h at 40 °C and then washed by deionized water until redundant PDA was removed. Subsequently, the PDA-treated support was dried in the oven at 50 °C.

### Molecular Dynamics Simulation

To capture the role of PIL on the diffusive ability of water under 2D confinement, we inserted the water, PIL, and NH_4_^+^ into the nanochannel constructed by GO. The functional groups in GO are mainly −COO^–^ and −OH, whose ratio to the carbon atom is *n*_COO_:*n*_OH_:*n*_C_ = 0.05:0.05:1. The numbers of water, PIL, and NH_4_^+^ are summarized in Table S1. Meanwhile, one PIL chain consists of 10 units of the cation as shown in Figure S14 in the MD simulations. The size of the GO sheet is 15.0 × 5.0 nm^2^, and a periodic boundary condition (PBC) is used in the *x*- and *y*-directions while an open boundary is applied in the *z*-direction.

The parameters of the bond, angle, dihedral, van der Waals interactions, and electrostatic interactions of PILs, GO, and NH_4_^+^ are described by the all-atom optimized potential for liquid simulations (OPLS-AA) force field. To accurately describe the electrostatic interactions of ILs, all charges were calculated *via* fitting the electrostatic potentials from the first principle calculations. The atomic charge of PIL is calculated based on the two-stage restraint electrostatic potential (RESP) method using the Gaussian 09 D.01 program package. The ionic geometry and atomic charge were obtained with the 6-311+G basis set for all elements in PILs. The rigidly extended simple-point-charge (SPC/E) model was used to describe the water molecules. The nonbonding interactions between different atoms in the system can be divided into electrostatic and van der Waals terms. The former one, the long-range Coulombic interaction, was computed by using the particle–particle–particle–mesh (PPPM) algorithm. The latter one was represented by using the 12–6 Lennard-Jones (LJ) potential, which was truncated at 1.2 nm. The Lorentz–Berthelot mixing rules were used to calculate the van der Waals interactions between different atomic species. The SHAKE algorithm was applied to hydrogen atom-related bonds to reduce high-frequency vibrations.

All the MD simulations in this work were performed using the large-scale atomic/molecular massively parallel simulator (LAMMPS). The carbon atoms in the GO sheet were fixed during the MD simulation to maintain the planar structure. The time step is 1.0 fs. After a 10 ns simulation, the GO/PIL/water system will reach the equilibrated state, where the final distance between two GO sheets is about 0.98 nm for PIL-**Tf**_**2**_**N**, PIL-C1, and PIL-C2. After the system was equilibrated, the MD simulations continue to run an additional 3.0 ns to collect the data to analyze the structure and calculate the self-diffusive coefficient. The molecular diffusion coefficient *D* is calculated from the molecular trajectories of water using Einstein’s definition that relates mean-square distance (MSD) to *D* as
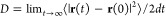
Here, *d* is the dimension of space for diffusion, *t* is the diffusing time, and ⟨...⟩ is the ensemble average that is implemented by averaging *D* obtained from 5 independent simulation runs.

### Characterizations

The concentration of dyes in the feed and permeate was measured by a UV–vis detector (GENESYS 150 UV–vis spectrometer). ^1^H NMR spectra were recorded at room temperature using a Bruker DPX-400 spectrometer operating at 400 MHz. DMSO-*d*_6_ was used as a solvent for the measurement. The morphology measurement of the membrane was performed by scanning electron microscopy (SEM) conducted on a JEOL 7000 instrument operated at 3 kV. Membranes were coated with a thin gold layer for 40 s before the examination. Powder X-ray diffraction (PXRD) patterns were collected on a Bruker AXS D8 Advance diffractometer (D8 Advance, Bruker) using Cu Kα radiation under 40 kV and 40 mA in the scan range 5–20° with the scan step of 0.05°. Attenuated total reflection FTIR spectroscopy was performed using a Vertex-70 spectrophotometer (Bruker) to characterize the functional group on the membrane. The membrane hardness was performed using a Nanoindentation G200 instrument (Agilent-U9880A) to characterize the mechanical strength. The nanoconfined space was characterized by low-field nuclear magnetic resonance (LF-NMR) (VTMR23-010 V-T, Suzhou Niumag Corporation).

### Dye Rejections by Nanofiltration

Our flat-sheet cross-flow nanofiltration device contains a membrane cell (the effective membrane area is 7.25 cm^2^), plunger pump, pressure gauge (1–8 bar), and solution vessel (Figure S22). The organic dye molecule (Evens blue) concentration of 100 mg/L in aqueous solution was used as the feed and pressurized with a plunger pump. As the Evens blue concentration was circulated, the permeate was collected at the same time. The permeance (*J*) was measured by the volume of the permeate sample in a certain collecting time and then calculated using the following equation:
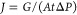
1where *G*, *A*, *t*, and *P* represent the volume of permeate (L), the effective area of the membrane (m^2^), the collecting time (h), and the operating pressure (bar), respectively.

The rejection of dye molecules was calculated by the following equation:
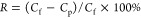
2where *C*_f_ and *C*_p_ represent the concentrations in the feed and the permeate, respectively.

## Supporting Information Available

Additional data and figures including chemical structures and characterization data, digital photograph, XPS analysis, ζ potentials, schematic of self-made pressured deposition device, AFM analysis, water permeance and dye (Evens blue) rejection, wet-state XRD patterns, cross-sectional SEM images, snapshot of equilibrium structure, comparison of separation performance, rejection of different dyes, comparison of COO^–^–GO@PIL**Tf**_**2**_**N**–AT separation performance, comparison of rejection and water permeance, schematic diagram of a flat-sheet cross-flow separation device, and the number of molecules in each component of the simulation systems (PDF)
