# Supplementary material for: “Mix-Then-On-Demand-Complex”: In Situ Cascade Anionization and Complexation of Graphene Oxide for High-Performance Nanofiltration Membranes
Source: ACS Nano. 2021 Feb 15;15(3):4440–9. doi: 10.1021/acsnano.0c08308 (PMC7992131; doi:10.1021/acsnano.0c08308)
Supplement: Supplementary file 1 — nn0c08308_si_001.pdf [file nn0c08308_si_001.pdf]

Electronic Supplementary Information

"Mix-Then-on-Demand-Complex": In-Situ Cascade Anionization and Complexation of Graphene Oxide for High-Performance Nanofiltration Membranes

Xiaoting Li[1,2], Yanlei Wang[3], Jian Chang[2], Hao Sun[1], Hongyan He[3], Cheng Qian[3], Atefeh Khorsand Kheirabad[2], Quan-Fu An[1], Naixin Wang[1]*, Miao Zhang[2]*, Jiayin Yuan[2]*

[1]Key Laboratory for Green Catalysis and Separation and Department of Environmental Chemical Engineering, Beijing University of Technology, Beijing, 100124, P. R. China.
Email: wangnx@bjut.edu.cn

[2]Department of Materials and Environmental Chemistry, Stockholm University, Stockholm, 10691, Sweden. Email: miao.zhang@mmk.su.se, jiayin.yuan@mmk.su.se

[3]Beijing Key Laboratory of Ionic Liquids Clean Process, State Key Laboratory of Multiphase Complex Systems, CAS Key Laboratory of Green Process and Engineering, Institute of Process Engineering, Chinese Academy of Sciences, Beijing 100190, China.

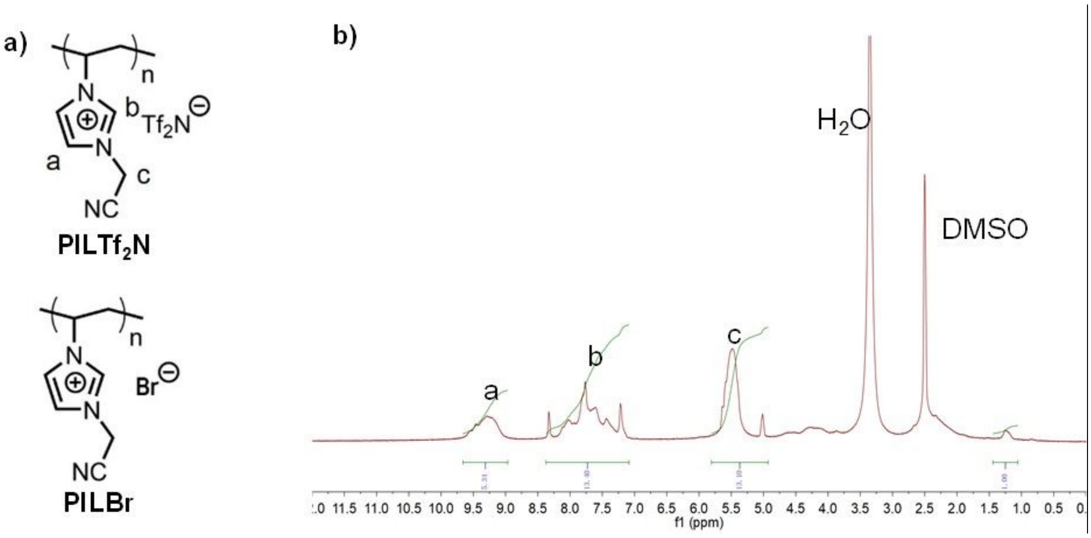

Figure S1. (a) Chemical structures of PIL**Tf_2N** and PIL**Br**; (b)^{1}H-NMR spectrum of PIL**Tf_2N** in DMSO-*d_6*.

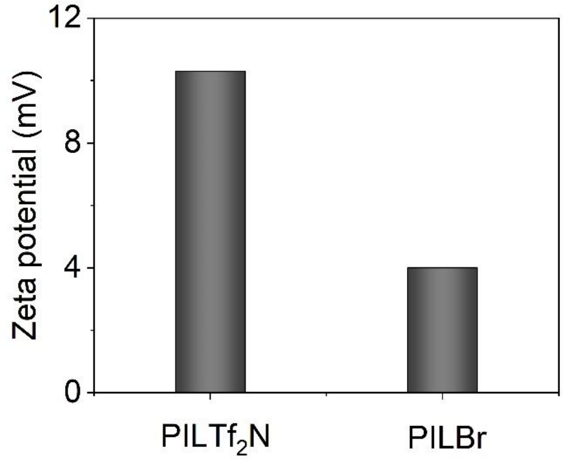

Figure S2. Zeta potentials of PIL**Tf_2N** in DMSO (+10.2 ± 1.0 mV) and PIL**Br** in DMSO (+4.0 ± 2.0 mV).

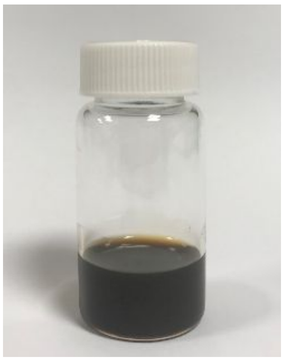

Figure S3. A digital photograph of a viscous COOH-GO dispersion in DMSO at a high concentration of 10 mg mL^{-1}. No precipitate was found at the bottom.

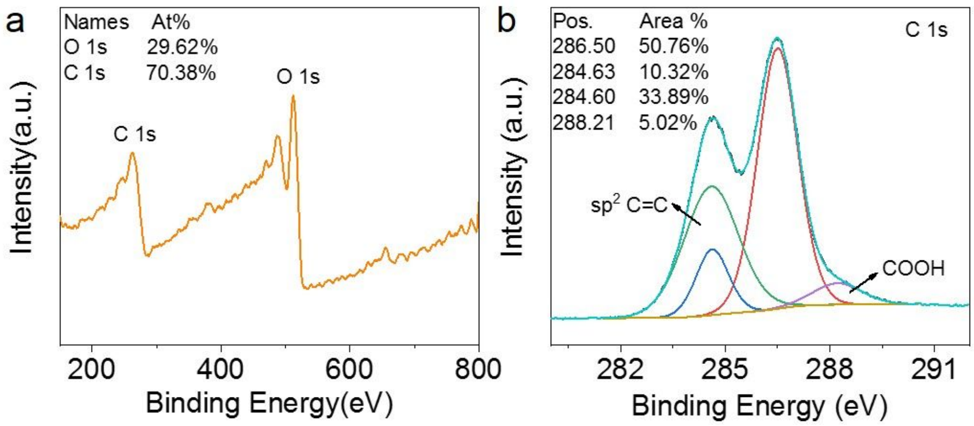

Figure S4. XPS analysis of COOH-GO powders.

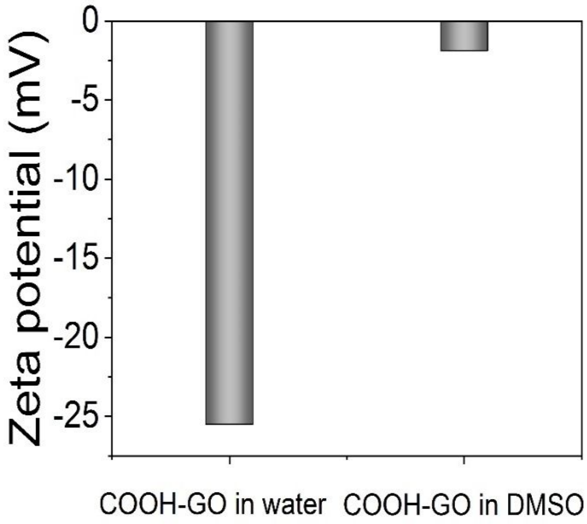

Figure S5. Zeta potentials of COOH-GO in water (-25.5 ± 1.0 mV) and DMSO (-1.8 ± 1.1 mV).

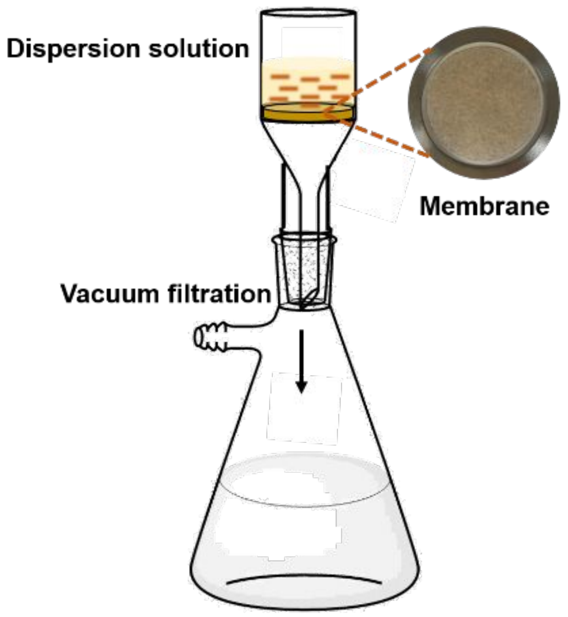

Figure S6. Schematic of self-designed pressured deposition device.

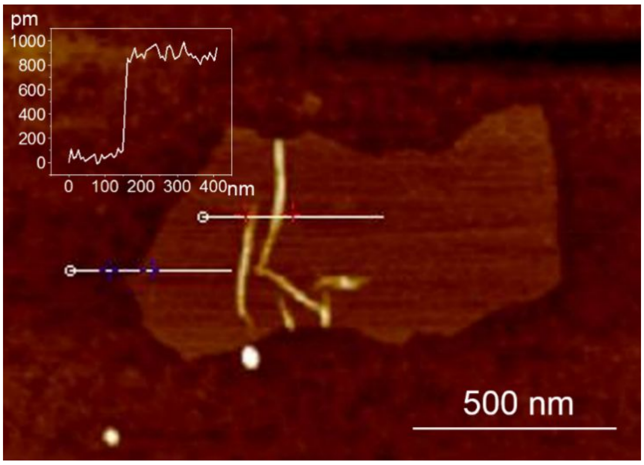

Figure S7. A representative AFM image of the GO nanosheets deposited on a mica surface.

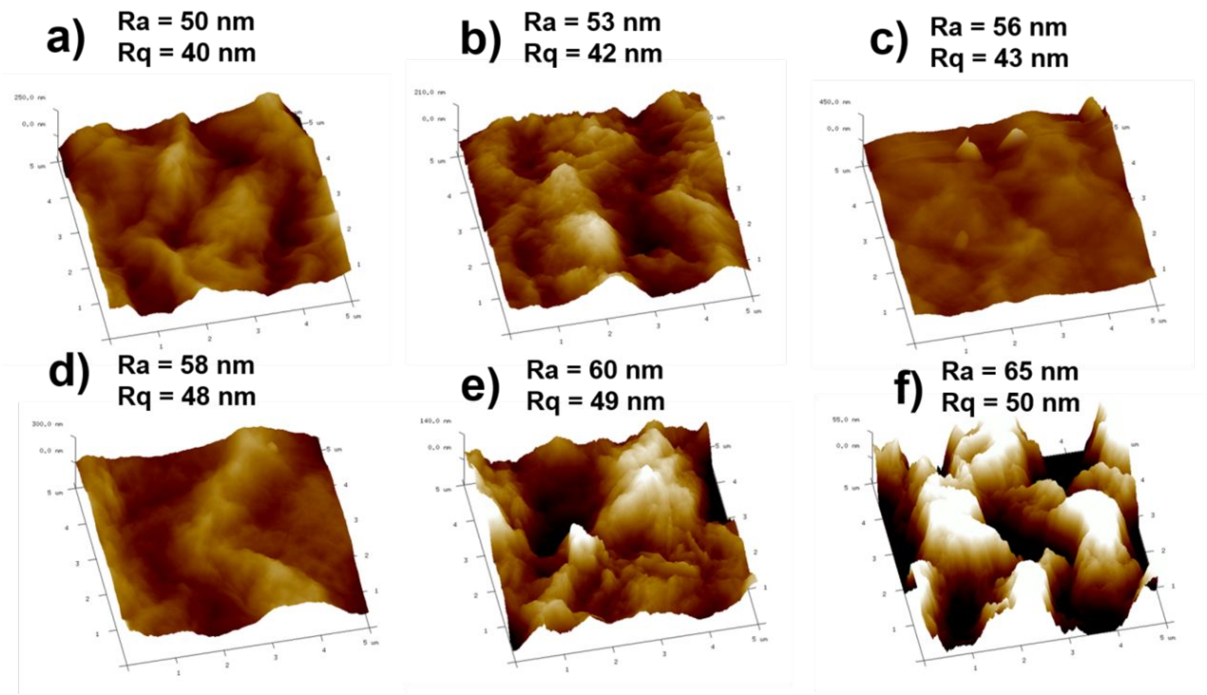

Figure S8. AFM phase and height images of a) PIL-free pristine COOH-GO membrane and b-f) COO^--GO@PIL**Tf$_2$N**-AT with different PIL**Tf$_2$N** concentrations (with regard to COOH-GO): b) 10%; c) 15%; d) 20%; e) 25%; 30%.

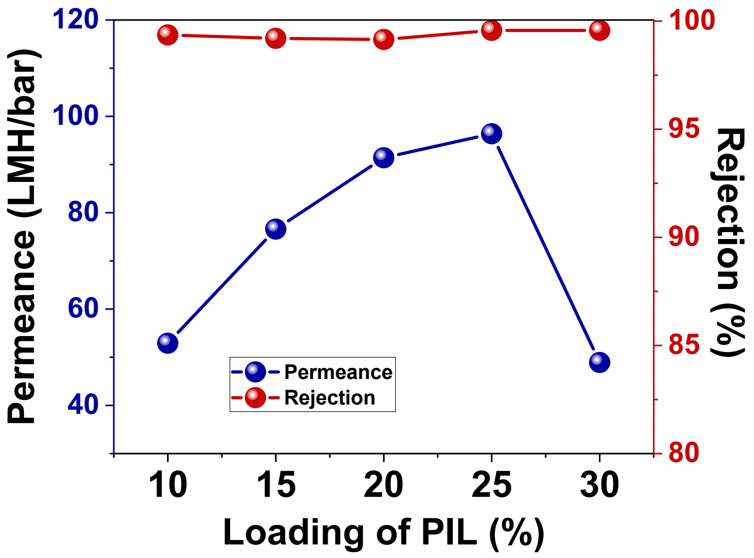

Figure S9. Water permeance and dyes (Evens blue) rejection of COO^--GO@PIL**Tf$_2$N**-AT with different PIL**Tf$_2$N** loading (with regard to COOH-GO).

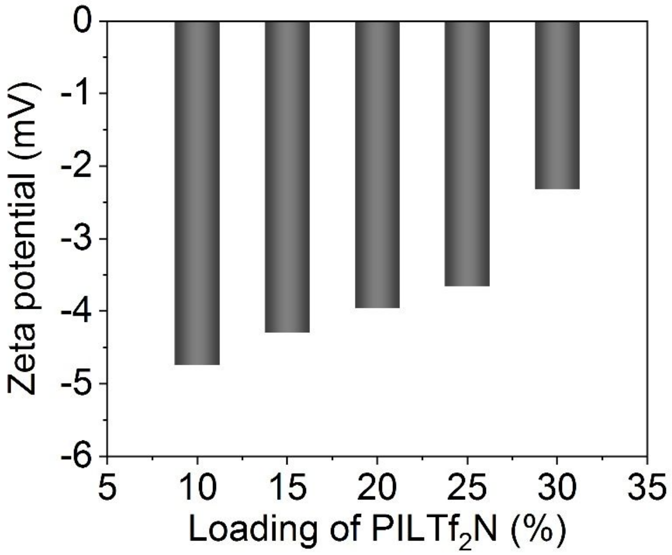

Figure S10. Zeta potentials of COO$^-$-GO@PIL**Tf$_2$N**-AT at different loading of PIL**Tf$_2$N** in DMSO.

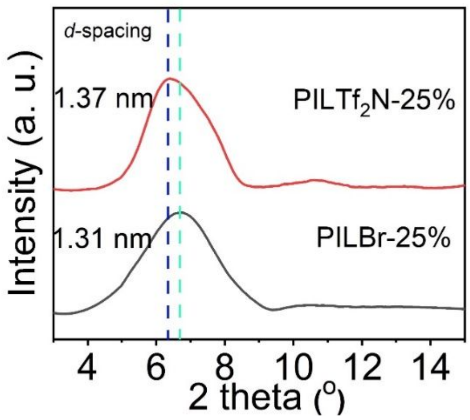

Figure S11. Wet-state XRD patterns of the COO$^-$-GO@PIL**Tf$_2$N**-AT and COO$^-$-GO@PIL**Br-**AT (at a polymer loading of 25%).

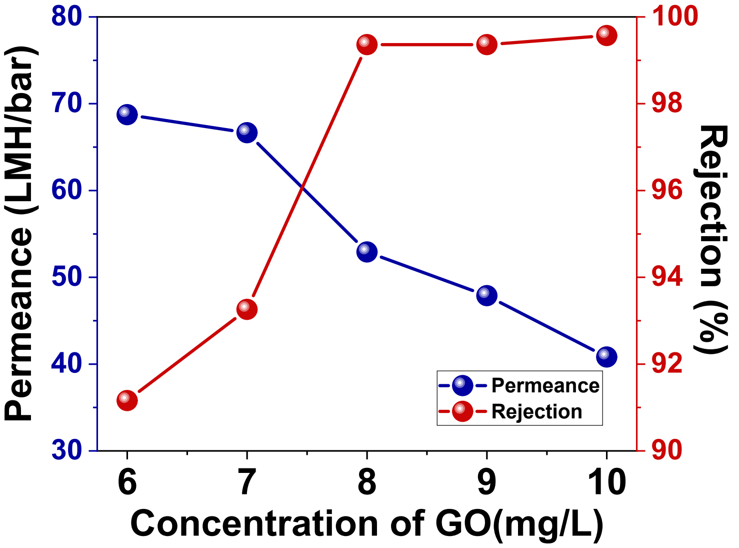

Figure S12. Water permeance and dyes (Evens blue) rejection of COO^--GO@PIL**Tf_2N**-AT at different GO concentration.

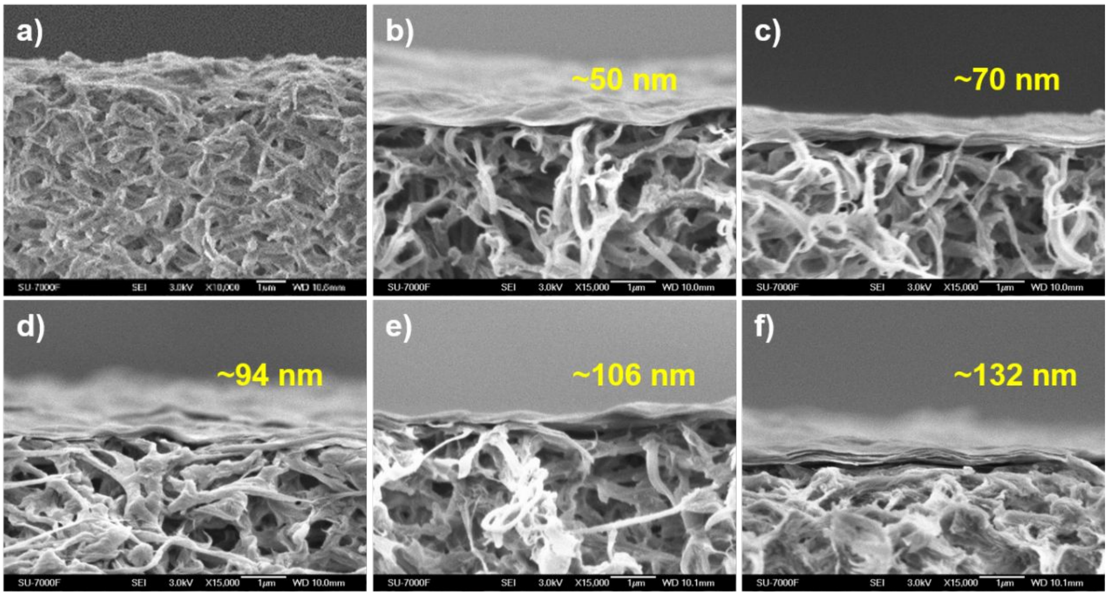

Figure S13. Cross-sectional SEM images of a) nylon-66 substrate; b-f) COO^--GO@PIL**Tf_2N**-AT with different GO concentration: b) 6 mg/L; c) 7 mg/L; d) 8 mg/L; e) 9 mg/L; f) 10 mg/L. The membrane thickness was determined by the average value of experimental data at five different locations on each sample.

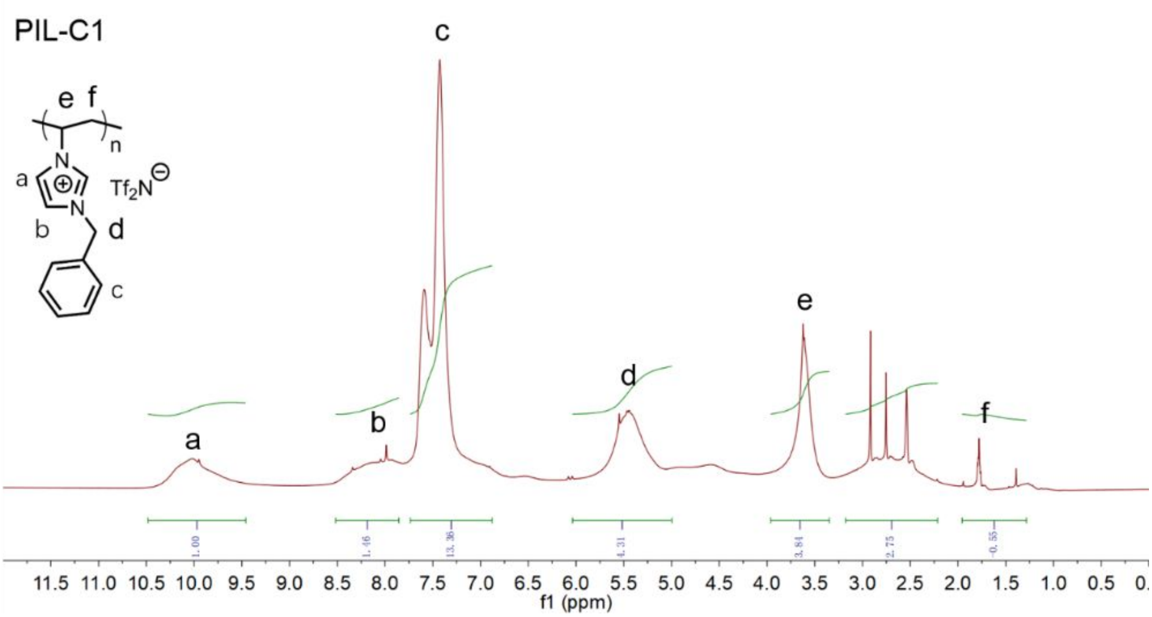

Figure S14. ^{1}H-NMR spectrum of PIL-C1 in DMSO-d_6.

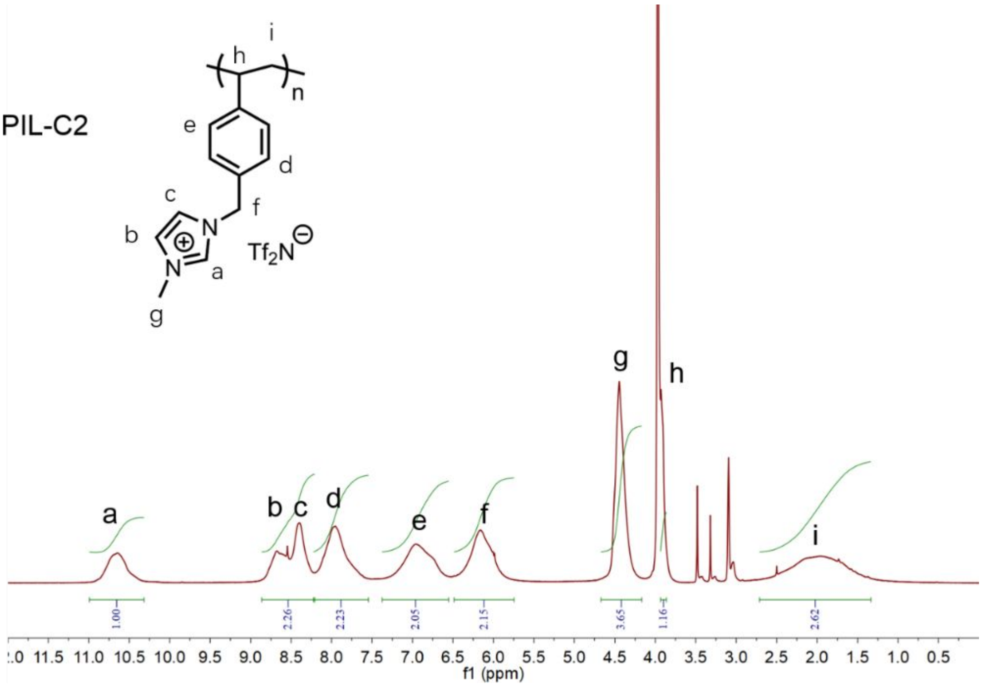

Figure S15. ^{1}H-NMR spectrum of PIL-C2 in DMSO-d_6.

Table S1. The number of molecules in each component of the simulation system.

PIL type	PIL	TFSI	Water	NH_4^+
PIL**Tf_2N**	2	20	300	60
PIL-C1	2	20	200	60
PIL-C2	2	20	200	60

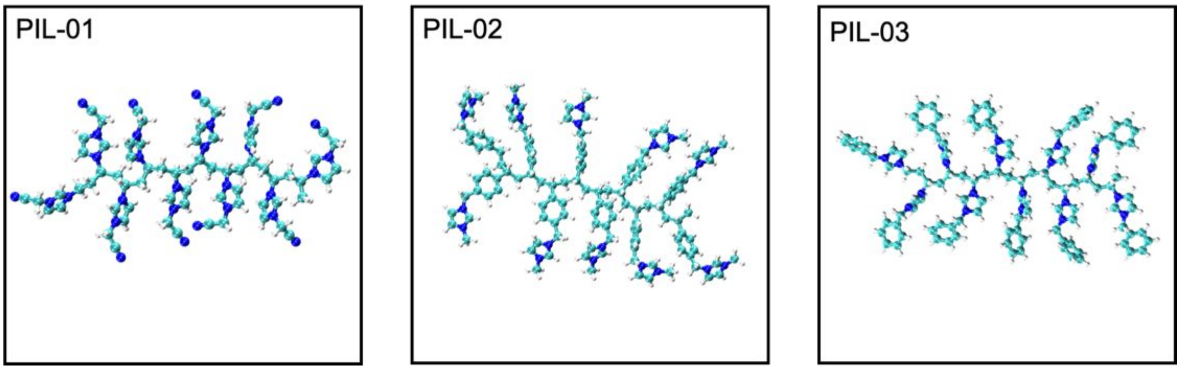

Figure S16. The snapshot of equilibrium structure of PIL cation chains in the nanochannels.

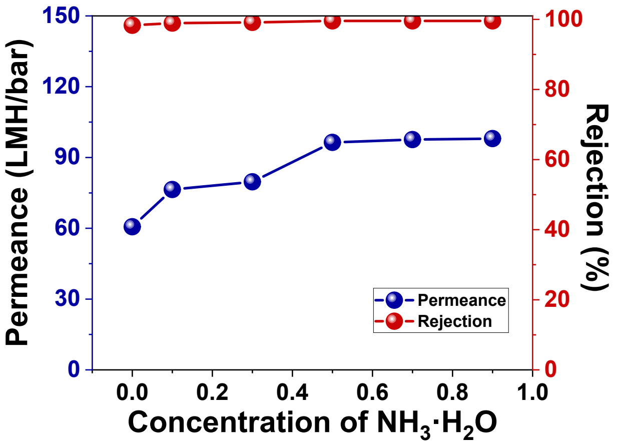

Figure S17. Water permeance and dyes (Evens blue) rejection of COO$^-$-GO@PIL**Tf$_2$N**-AT at different ammonia concentration.

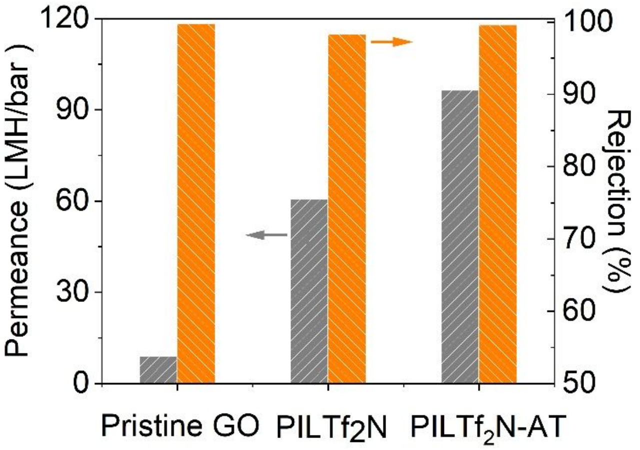

Figure S18. Comparison of separation performance for pristine PIL-free GO membranes, COOH-GO@PIL**Tf$_2$N** (without ammonia treatment), and COO$^-$-GO@PIL**Tf$_2$N**-AT (with ammonia treatment).

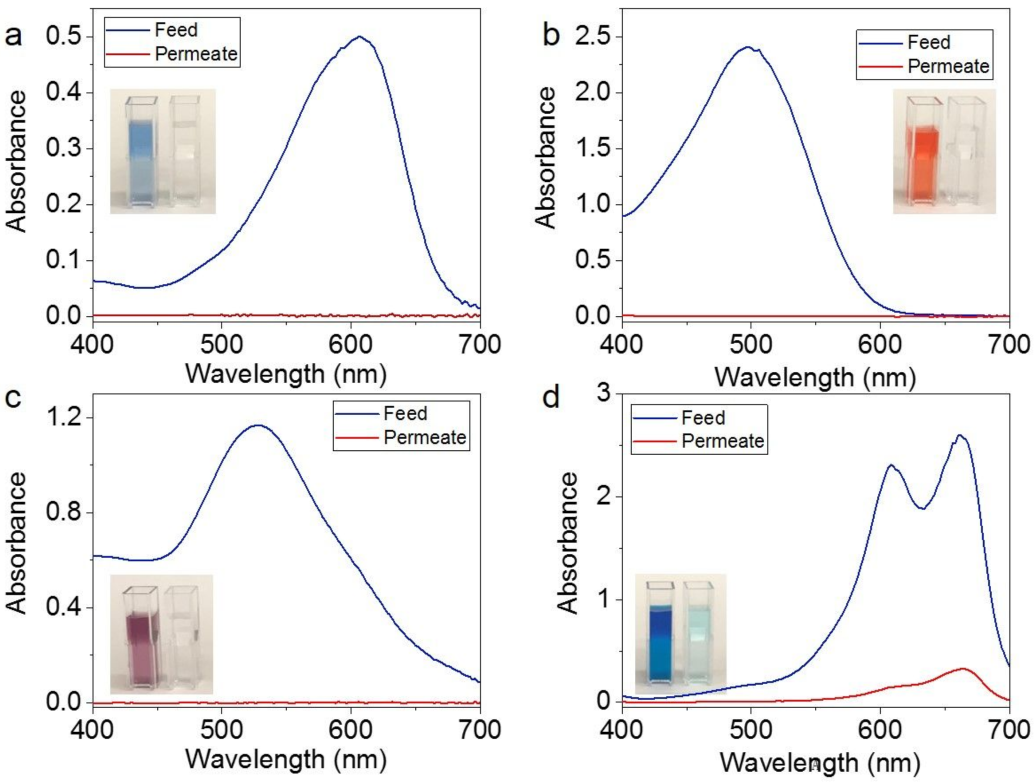

Figure S19. Rejection of different dyes for COO$^-$-GO@PIL**Tf$_2$N**-AT, a) Evans blue; b) Congo red; c) Eriochrome black T; d) Methylene blue.

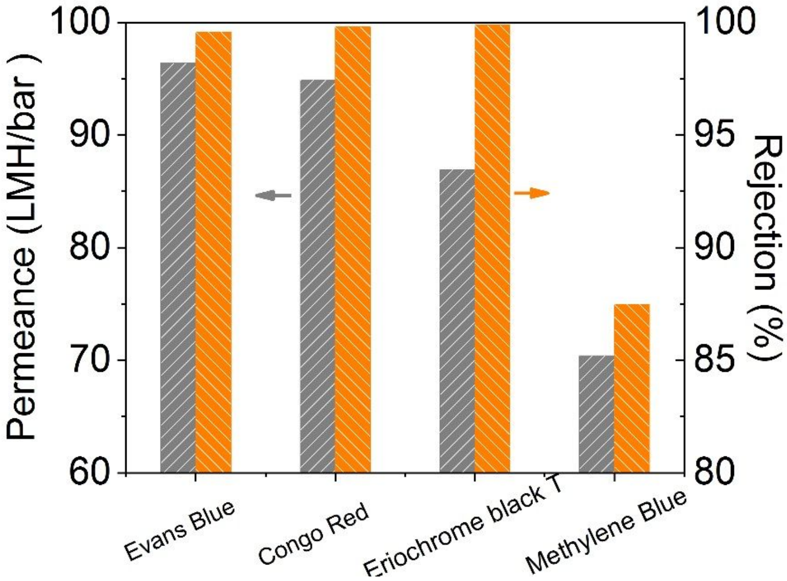

Figure S20. Comparison of COO$^-$-GO@PIL**Tf$_2$N**-AT separation performance for different dye molecules.

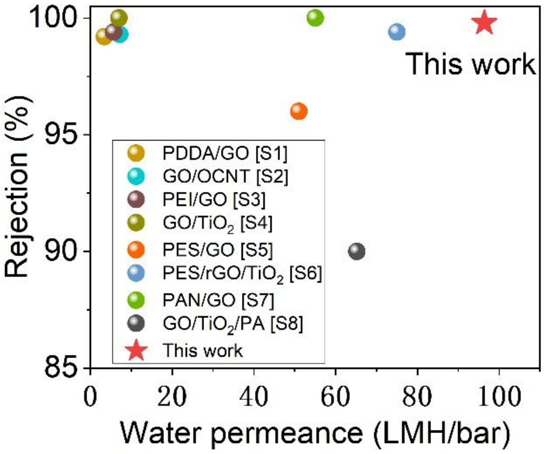

Figure S21. Comparison of rejection and water permeance for GO-based hybrid membranes reported in literature with the GO-based membrane in this work.

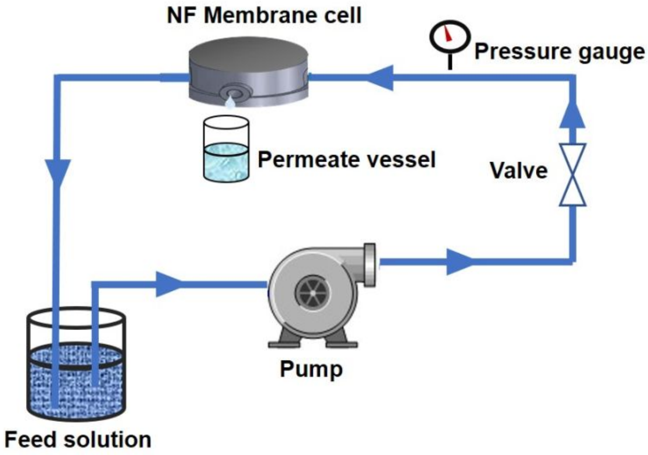

Figure S22. Schematic diagram of a flat-sheet cross-flow separation device.

References

[1] L. Wang, N. Wang, J. Li, W. Bian, S. Ji. Layer-by-Layer Self-assembly of Polycation/GO Nanofiltration Membrane with Enhanced Stability and Fouling Resistance. *Sep. Purif. Technol.* **2016**, *160*, 123−131.

[2] H. Kang, J. Shi, L. Liu, M. Shan, Z. Xu, N. Li, H. Lv, X. Qian, L. Zhao. Sandwich Morphology and Superior Dye-Removal Performances for Nanofiltration Membranes Self-assemblied *via* Graphene Oxide and Carbon Nanotubes. *Appl. Surf. Sci.* **2018**, *428*, 990−999.

[3] N. Wang, S. Ji, G. Zhang, J. Li, L. Wang. Self-assembly of Graphene Oxide and Polyelectrolyte Complex Nanohybrid Membranes for Nanofiltration and Pervaporation *Chem. Eng. J.* **2012**, *213*, 318−329.

[4] C. Xu, A. Cui, Y. Xu, X. Fu. Graphene Oxide–TiO_2 Composite Filtration Membranes and Their Potential Application for Water Purification. *Carbon* **2013**, *62*, 465−471.

[5] S. Zinadini, A. A. Zinatizadeh, M. Rahimi, V. Vatanpour, H. Zangeneh. Preparation of a Novel Antifouling Mixed Matrix PES Membrane by Embedding Graphene Oxide Nanoplates *J. Membr. Sci.* **2014**, *453*, 292−301.

[6] M. Safarpour, V. Vatanpour, A. Khataee. Preparation and Characterization of Graphene Oxide/TiO_2 Blended PES Nanofiltration Membrane with Improved Antifouling and Separation Performance *Desalination* **2016**, *393*, 6−78.

[7] Z. Qiu, X. Ji, C. He. Fabrication of a Loose Nanofiltration Candidate from Polyacrylonitrile/Graphene Oxide Hybrid Membrane *via* Thermally Induced Phase Separation. *J. Hazard. Mater.* 2018, 360, 122−131.

[8] Y. Gao, M. Hu, B. Mi. Membrane Surface Modification with TiO_2–Graphene Oxide for Enhanced Photocatalytic Performance. *J. Membr. Sci*. **2014**, *455*, 349−356.
